# Patient experience preparing for prostate cancer radiotherapy

**DOI:** 10.1016/j.tipsro.2025.100306

**Published:** 2025-02-17

**Authors:** S.E. Alexander, J. Selous-Hodges, A. Araujo, L. Booth, L. Delacroix, E. Garrad, A. Gordon, C. Graham, A. Guerra, C. Gulyaeva, C. Ockwell, S. Shire, U. Oelfke, H.A. McNair, A.C. Tree

**Affiliations:** a The Royal Marsden NHS Foundation Trust and the Institute of Cancer Research, United Kingdom; b The Royal Marsden NHS Foundation Trust, United Kingdon; c The Joint Department of Physics, The Royal Marsden Hospital and the Institute of Cancer Research, United Kingdom

**Keywords:** Patient experience, Patient preparation for radiotherapy, Bladder and rectal preparation, Prostate cancer

## Abstract

**Introduction:**

Bladder and rectal preparation regimes for prostate cancer (PCa) radiotherapy (RT) can heighten anxiety before and during RT. Patient’s perception of RT preparation is under-represented in the literature. To address this gap, patient’s experience of preparation with respect to understanding, comfort, anxiety, effectiveness and impact on daily life was examined.

**Materials and methods:**

A novel patient preparation survey was created and validated, it contained 12 original questions related to general, bladder and rectal preparation. Plus, the Patient Health Questionnaire 4 (PHQ4) and question 15 of the Expanded Prostate Cancer Index Composite (EPIC).

Eligible patients were individuals referred for prostate or prostate bed +/− pelvic lymph node RT from March-May 2024. Surveys were issued immediately after the patient’s planning scan, those completing the survey at this timepoint were asked to repeat it in their first and final weeks of RT.

**Results:**

The survey was completed by 103/125 eligible patients at their planning scan, 47/103 in the first and 52/103 in the final week of RT. Perception of preparation was largely positive. For general and bladder preparation positive question response rate ranged from 55 to 98 % and negative from 0 to 26 %. Rectal preparation response rate was 59–100 % positive and 0–35 % negative. Difficulty maintaining a full bladder and using enemas was greatest at the end of RT.

No significant difference in experience was found for participants using or not using enemas for preparation. Anxiety and depression (PHQ4) affected 12–13 % of respondents, and significantly more patients reported bowel toxicity (EPIC), in the last week of RT compared to earlier timepoints.

**Conclusion:**

The authors conclude that the preparation needs of their patients are well met. However, a considerable number did find preparation difficult, disruptive and ineffective, more so at the end of treatment. Further qualitative analysis of patient’s experience is needed to better understand why individuals experience varies.

## Highlights

• Patient’s experience of preparation for prostate cancer RT was largely positive.
• A considerable number found preparation difficult, disruptive and ineffective, more so at the end of treatment.
• Difficulty maintaining a full bladder and using enemas was greatest at the end of RT.
• No significant difference in experience was found for patients using or not using enemas for preparation.
• Anxiety and depression (PHQ4) affected 12-13% of respondents during RT.

## Introduction

Preparing for radiotherapy (RT) can be particularly stressful [1] due to information overload [2]. For individuals requiring RT for localised prostate cancer (PCa), recommended preparation involves following strict bladder and rectal preparation regimes [3]. Anecdotal evidence tells us that having to adhere to a rigid preparation regime heightens patient’s anxieties before and during PCa RT, however, the patient voice is under-represented in this area [4].

**Fig. 1:**
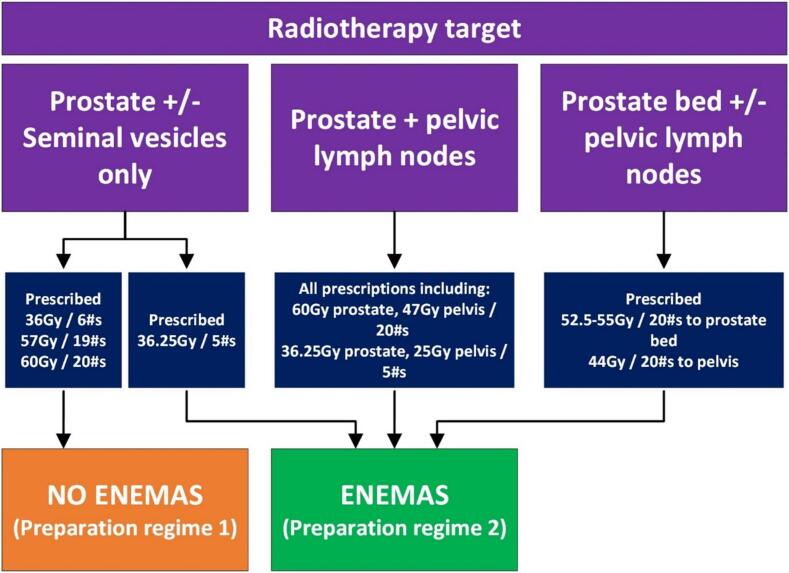
Patient groups using/not using enemas for prostate cancer radiotherapy preparation.

Having examined the impact of large rectal volumes on intrafraction prostate motion, and reported no association [5], in October 2023 routine rectal preparation for a subset of patients undergoing PCa RT was ceased. Preparation stratification criteria is presented in Fig. 1. The updated regime reduced information and preparation burden, so was anticipated to improved patient experience. However, no existing questionnaires were available in the literature to evaluate this impact. This led researchers to develop a questionnaire to capture patient’s experience preparing for PCa RT, with respect to understanding, comfort, anxiety, effectiveness and impact on daily life.

## Materials and methods

### Development and validation of the patient preparation survey

A novel patient preparation survey was created to establish patient’s experience preparing for PCa RT with respect to:

• Understanding, comfort, anxiety, effectiveness and impact on daily life

Version one of the survey was informed by the multi-disciplinary research team’s clinical experience preparing and caring for people during PCa RT. This survey was reviewed by three patient and public contributors whose feedback was incorporated to improve question order, wording and clarity.

Version two of the survey was sent via e-mail to six therapeutic radiographers specialising in PCa or research methods, working across multiple NHS hospitals, to rate the content validity index (CVI) [6] of each question, and appraise survey language, clarity of words/terms used and breadth of questions. For each question CVI was judged on a four-point scale; ‘1’ = not relevant, ‘2’ = somewhat relevant, ‘3’ = quite relevant and ‘4’ = highly relevant. Answers were dichotomised to ‘not relevant’ (‘1’ and ‘2’) and ‘relevant’ (‘3’ and ‘4’). When an expert found an item to be relevant, it was given a CVI of ‘1’ and when it was not, CVI was ‘0’. CVI was evaluated on item-level and calculated by summing the number of experts rating the item as relevant (‘1’) divided by the number of experts. With six experts a minimum CVI of 0.78 was required for inclusion, signifying that the majority agree the question is relevant [6]. All questions in the final version of the survey achieved a CVI score of 1.

The final survey (Supplementary material A) is composed of 12 original questions. Patients on preparation regime 1 (no enemas) (Table 1) complete questions 1–8 while those following regime 2 (with enemas) complete questions 1–12.

**Table 1:** Preparation regimes in clinical practice.

Regime	Rectal preparation at planning scan	Rectal preparation during radiotherapy	Bladder preparation at planning scan and during radiotherapy
Preparation regime 1 (new October 2023)	None	None	A comfortably full bladder, 350 ml of water 30–60 min prior to CT and treatment.
Preparation regime 2 (original)	Micro-enema for 2 days prior to and 2 h before the CT scan	Micro-enema for 2 days prior to and 2 h before treatment (continued daily for half or all treatments depending on duration)	A comfortably full bladder, 350 ml of water 30–60 min prior to CT and treatment.

Measurement of psychological distress and bowel symptoms were incorporated into the survey, as they can impact patient experience [1], [2], [7]. All patients complete the Patient Health Questionnaire 4 (PHQ4) and question 15 of the Expanded Prostate Cancer Index Composite (EPIC). PHQ4 is composed of four questions which give an ultra-brief measurement of core depression and anxiety symptoms [8], [9]. PHQ4 is scored from 0 to 12, with the following total corresponding to categories of psychological distress:

• 0–2, none
• 3–5, mild
• 6–8, moderate
• 9–12, severe

The EPIC questionnaire is designed to evaluate patient function and bother after PCa treatment, question 15 of EPIC is composed of six questions for quick assessment of bowel urgency, frequency, diarrhoea, incontinence, bleeding and pain [10], [11]. Each question is scored 0–4 from “no-problem” to “big problem”. No scoring guide is given for question 15 of EPIC in isolation, so the researchers set the following total score criteria:

• 0, no problem
• 1–3, very small problem
• 4–6, small problem
• >6, moderate to big problem

The final survey was reviewed and approved by the hospitals service evaluation committee (Ref No: SE134, patient experience of preparation protocols for prostate cancer radiotherapy) in January 2024.

### Participant sampling and eligibility

Paper surveys were given to all individuals having a radical radiotherapy planning scan for PCa from March-May 2024 inclusive. This included those referred for radiotherapy to their prostate and seminal vesicles or prostate bed, with or without pelvic nodal irradiation. A three-month recruitment period was deemed sufficient to capture a sample representative of the local patient population, based on records from the prior six months detecting 40 to 50 eligible patients per month. A minimum requirement for 100 completed surveys was set.

Patients included followed one of two alternative preparation regimes (Table 1) depending on their RT fractionation and target volume (Fig. 1).

Completion of the survey was voluntary and functioned as consent to present individuals anonymised responses, as stipulated in the covering letter. Participant name and date of birth was acquired to facilitate distribution of follow-up surveys, to examine change in experience over time. Each participant completing the survey at their planning scan, was given the same survey at two further timepoints; first and final week of radiotherapy.

### Data analysis

Completed surveys were stored securely and transcribed from paper into a protected Microsoft excel workbook by a team of six therapeutic radiographers. Data was transcribed in batches, with each batch input by one radiographer and independently checked by another.

Data was analysed in four groups by theme: questions 2–7 relating to general and bladder preparation, questions 9–12 relating to bowel preparation, PHQ4, and question 15 of EPIC.

For questions 2–7 and 9–12 the 10-point Likert scale used varied in response direction depending on the question. Specifically, a score of 10 indicated a positive response for questions 2, 5, and 9 but a negative response for questions 3, 4, 6, 7, 10, 11, and 12. To aid clarity when presenting the results questions where a score of 10 indicated a negative response were inverted e.g. 10 became 1, 9 became 2, 8 became 3 etc… so that for all questions total scores relate as follows:

• 1–2, most negative
• 3–4, negative
• 5–6, neutral
• 7–8, positive
• 9–10, most positive

Descriptive statistics were used to analyse this data plus PHQ4 and EPIC results.

Question responses from participants following preparation regimes 1 and 2 were compared. Data was tested for normality and a Mann-Whitney test run for questions 2–7, PHQ4 and question 15 of EPIC using GraphPad PRISM (Boston, USA), a significant *p* value of 0.05 was set.

For complete datasets, i.e. three complete surveys for a participant, the responses between timepoints were analysed for question 2–7, 9–12, PHQ4 and EPIC differences. Using GraphPad PRISM, data was tested for normality, a Friedman test run to assess for significant difference across the three timepoints, followed by Dunn’s test for pairwise comparison. A significant *p* value of 0.05 was set.

The final question allowed for participants free text comments. Free text comments were analysed for each timepoint independently by two therapeutic radiographers. Comments were assessed as positive, negative and neutral. Key themes were identified from the free text by each reviewer, who then collaborated to agree the final themes.

## Results

The survey was given out at the pre-radiotherapy planning scan appointment from March 4th to May 31st, 2024, during which time 103 of 125 eligible patients participated. Completed survey numbers dropped for timepoint two and three, a full complement of completed surveys, from each timepoint, was available for 28/103 patients (Table 2).

**Table 2:** Tally of completed surveys per timepoint, and number per preparation regime followed.

Timepoint	Planning scan	First week of RT	Final week of RT
Total number of completed surveys	103	47/103 (46 %)	52/103 (50 %)
Number of surveys completed by patients following regime 1	64/103 (62 %)	29/47 (62 %)	32/52 (62 %)
Number of surveys completed by patients following regime 2	39/103 (38 %)	18/47 (38 %)	20/52 (38 %)
Number of patients completing the survey at all 3 timepoints	28/103 (27 %)		

Most patient’s experience of preparation was positive, results generated using all completed surveys (n = 202) are presented in Fig. 2. For questions 2–7 (Fig. 2A), relating to general and bladder preparation, positive response rate per question (a score of 7–10) ranged from 55 to 98 %. The most positive response rate was for question two, “I understood how to prepare for my radiotherapy scan/treatment”, this scored 96 % at the planning scan and 98 % at both first and final weeks of RT. Question five “I knew how to prepare my bladder for my radiotherapy scan/treatment” was the only other question to score a ≥ 80 % positive response rate for all timepoints. High positive scores for these two questions indicates that patients were well informed on how to prepare.

**Fig. 2A:**
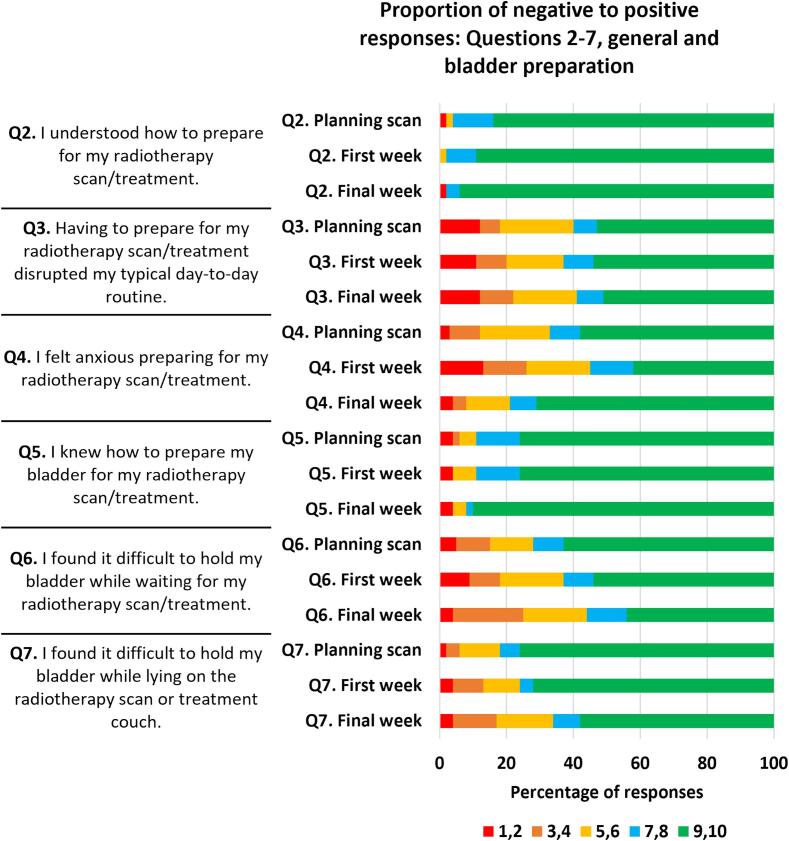
Proportion of negative to positive responses for questions 2–7. Score of 1–2 = most negative, 3–4 = negative, 5–6 = neutral, 7–8 = positive. 9–10 = most positive.

The rate of negative responses (a score of 1–4) ranged from 0 to 26 %. The most negative response rate was for question 4 “I felt anxious preparing for my radiotherapy scan/treatment”, in the first week of RT. Three additional responses triggered a negative response rate ≥20 %:

• Question three, “having to prepare for my radiotherapy scan/treatment disrupted my typical day-to-day routine”, first week of RT.
• Question three, final week of RT.
• Question six, “I found it difficult to hold my bladder while waiting for my radiotherapy scan/treatment”, final week of RT.

These negative responses tell us that although patients knew how to prepare, for some preparation inflicted anxiety, disruption and discomfort. A trend for negative response rates to increase from planning scan to final treatment was seen for questions six and seven. The negative response rate for question six was 15, 18 and 25 % for planning scan, first and final week of RT. The negative response rate for question seven, “I found it difficult to hold my bladder while lying on the radiotherapy scan or treatment couch” was 6, 13 and 17 % for planning scan, first and final week of RT. This tells us that as treatment progressed more patients found managing a full bladder difficult.

Neutral response rate (a score of 5–4), ranged from 0 to 22 %. Question three, relating to preparation disrupting day-to-day routine, scored the most neutral responses across all three timepoints.

For questions 9–12 (Fig. 2B), relating to rectal preparation, positive response rate per question ranged from 59 to 100 %. Akin to the first set of questions, the most positive response rate related to patients understanding of how to prepare. Question nine, “I knew when to use the enemas I was given” scored 87 %, 100 % and 95 % positive responses for planning scan, first and final weeks of RT respectively. No other questions in this section scored ≥80 % positive responses across all three timepoints.

**Fig. 2B:**
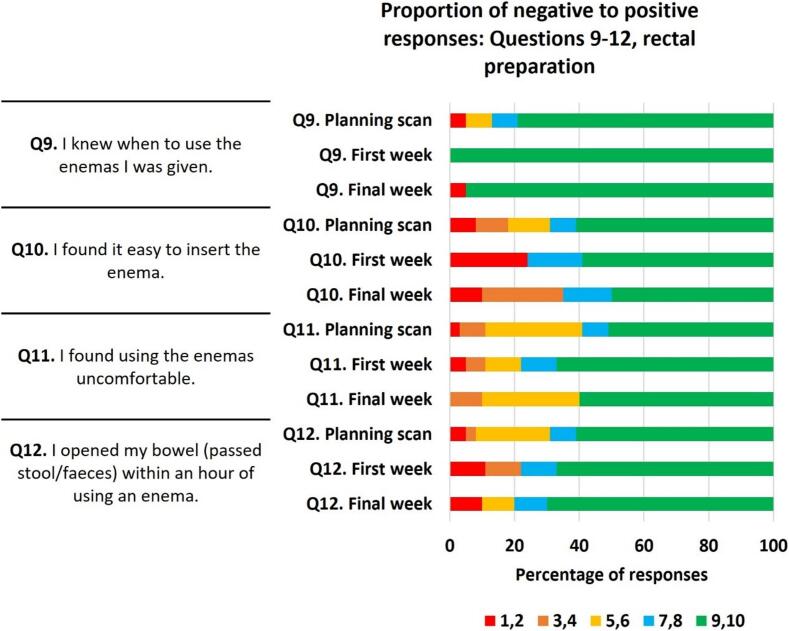
Proportion of negative to positive responses for questions 9–12. Score of 1–2 = most negative, 3–4 = negative, 5–6 = neutral, 7–8 = positive. 9–10 = most positive.

Negative response rate ranged from 0 to 35 %. The most negative response rates were for question ten, “I found it easy to insert the enema”, scoring 18 %, 24 % and 35 % at planning scan, first and final RT respectively, meaning more patients found enema insertion difficult as treatment progressed. One additional response triggered a negative response rate ≥20 %, question twelve, “I opened my bowel (passed stool/faeces) within an hour of using an enema”, first week of RT. Showing variability in enema effectiveness at an individual level.

Neutral response rate (a score of 5–4), ranged from 0 to 30 %. Question eleven “I found using the enemas uncomfortable” triggered the most neutral responses.

PHQ4 and question 15 of EPIC results, for all responses, are presented in Table 3.

**Table 3:** PHQ4 and EPIC results per timepoint.

PHQ4 total score	Number of respondents *planning scan*	Number of respondents *first week*	Number of respondents *final week*
<3 (normal)	90/103 (87 %)	41/47 (87 %)	46/52 (88 %)
3–5 (mild)	6/103 (6 %)	5/47 (11 %)	5/52 (10 %)
6–8 (moderate)	6/103 (6 %)	1/47 (2 %)	1/52 (2 %)
9–12 (severe)	1/103 (1 %)	0	0

**Question 15 of EPIC score**	**Number of respondents *planning scan***	**Number of respondents *first week***	**Number of respondents *final week***

0 (no problem)	61/103 (59 %)	31/47 (66 %)	15/52 (29 %)
1–3 (very small problem)	29/103 (28 %)	7/47 (15 %)	15/52 (29 %)
4–6 (small problem)	7/103 (7 %)	6/47 (13 %)	11/52 (21 %)
>6 (moderate problem)	6/103 (6 %)	3/47 (6 %)	11/52 (21 %)

Evaluation of responses for each timepoint, found no significant difference in question 2–7, PHQ4 and question 15 of EPIC scores for participants using or not using enemas as part of their preparation.

**Fig. 3:**
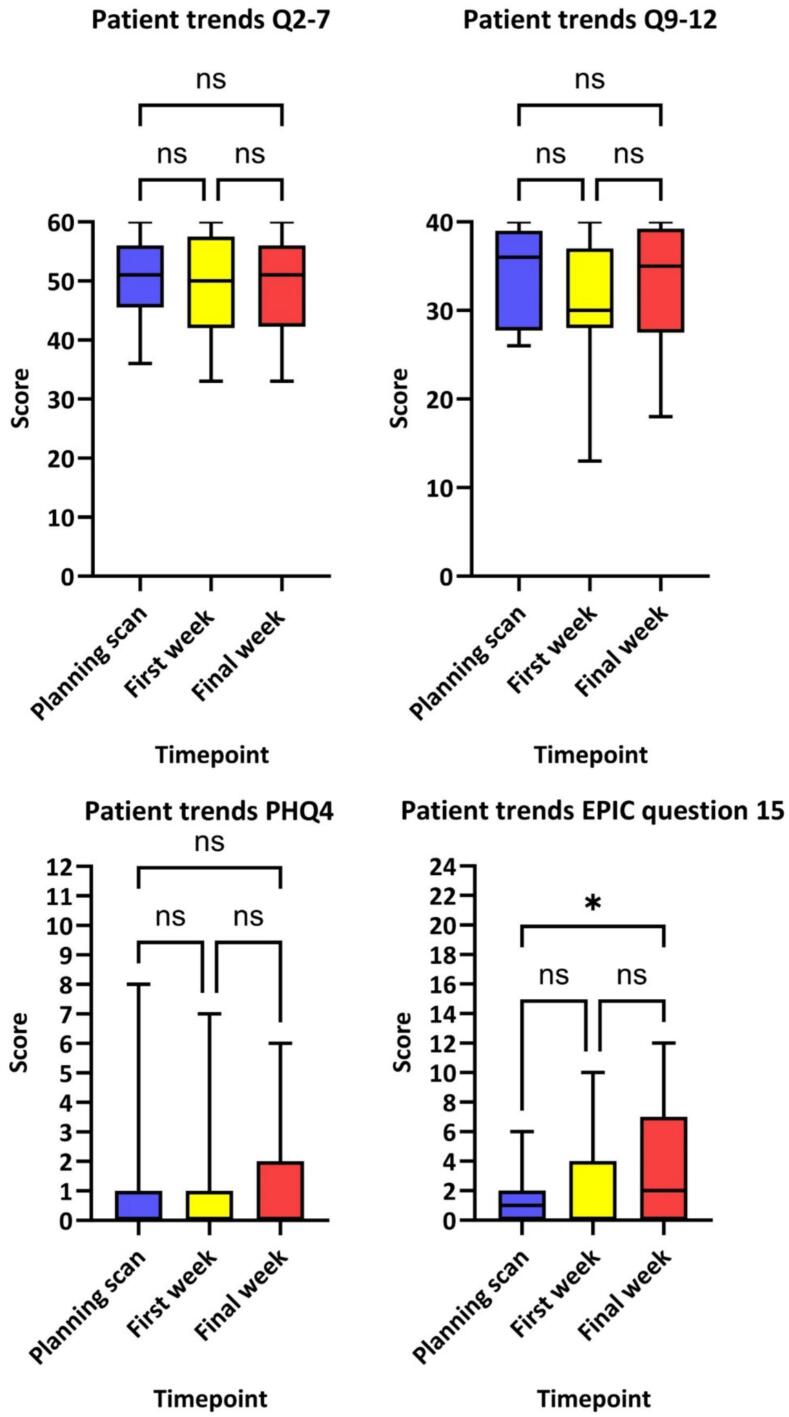
Results for the 28 participants who completed the questionnaire at all timepoints, for each group of questions. The Y-axis “score” relates to the highest score a patient could have obtained for the group of questions investigated. For example, “patient trends Q2-7” is composed of 6 questions, each with a maximum score of 10, as the 6 questions are being examined under one theme the total score is 60 (6 × 10). ns = not significant, * = p < 0.05.

Fig. 3 presents the results for the 28 participants who completed the questionnaire at all timepoints. The only significant difference seen was for the question 15 of EPIC score, between planning CT and final week (*p = 0.04*), representing an increase in bowel toxicity with treatment duration.

Free text comments were written by 45/103, 23/47 and 31/52 patients for the planning scan, first and final week of RT timepoints respectively. Eighty-one percent of comments (80/99) were positive, 10 % (10/99) neutral and 9 % negative (9/99). Thematic analysis of free text comments identified four key themes (Table 4). Themes 1–3 were positive, theme 4 expressed some concerns with the preparation process.

**Table 4:** Free text themes, the timepoints they appear and three examples per theme.

Key theme	Survey timepoint expressed	Examples
1. Clear guidance/a well explained process	All 3	*“Very clear instructions, written and verbal” (planning scan)* *“I was given clear instruction at every aspect of my treatment” (first week)* *“The details for prep were very comprehensive and clear…” (final week)*
2. Professional and supportive staff	All 3	*“Staff extremely empathetic and constantly making me feel at ease” (planning scan)* *“The radiographers who assisted me on my first appointment were extremely understanding and explained every step. This helped lower any anxiety I may have felt during my initial treatment” (first week)* *“Please relay my gratitude for the professional and sensitive way in which the staff carried out the procedures” (final week)*
3. Positive treatment experience	All 3	*“First class treatment” (planning scan)*
		*“Pleasantly surprised, I was expecting to feel something during the therapy but nothing at all” (first week)* *“…I am very happy and satisfied with my treatment offered” (final week)*
4. Bladder and rectum symptoms/preparation	First and final week of treatment	*“Constipation stress induced” (first week)* *“…progressively, through treatment I have developed difficult in passing water” (final week)* *“Enemas only used for the first couple of appointments then not used…had no issues with empty bowel by natural body function” (final week)*

## Discussion

A key survey finding is that patients largely understood how to prepare for PCa RT and were not overwhelmed by the information received. The positive response rate of 96 % for general preparedness for RT, at the planning scan, aligns favourably with the National Cancer Patient Experience Survey (2023), where 88.8 % of RT patients stated they “completely had enough understandable information” [12]. The authors attribute the high scores achieved to the comprehensive three-pronged approach taken with respect to information giving. All PCa patients for RT, at the hospital, attend a group preparation seminar [13], have a doctor-led face-to-face consent appointment, and a follow-up urology-specialist therapeutic radiographer-led telephone call, prior to their planning scan. This approach enables information to be given in stages, aligned with patient’s changeable information needs [14].

The telephone call is a new addition to the preparation process, which may have contributed to improved patient preparation rates, compared to prior evaluation [13]. Prior evaluation (2017–19) found 73 % of PCa patients felt completely prepared for RT having attended a preparation seminar and consent appointment. Direct comparison is however complicated by different scoring systems. Telephone-based interventions are reported as a valued source of reassurance, continuity of care and practical information giving for persons with cancer [15] saving time and travel expense [16], [17].

Despite most patients having a positive experience of bladder and rectal preparation, a considerable number did find preparation difficult, disruptive and ineffective. Acute genitourinary toxicity, including urgency and frequency peak at the end of RT [18], [19], it was therefore no surprise to find an increase in patient discomfort managing a full bladder for RT, as treatment progressed. We did not track patients’ bladder volumes, but a trend for bladder volume to reduce as PCa RT progresses is reported elsewhere [20], [21]. Empty bladder protocols are followed by some hospitals [22], [23] but associated patient comfort and experience data was not found. The authors bladder preparation preference remains a comfortably full bladder, to satisfy bladder dose constraints and displace small bowel from the treatment field [21], [24], [25], [26], [27]. Aligned with this, future efforts are focussed on improving patient comfort and experience while maintaining a comfortably full bladder using a daily hydration prescription, as opposed to strict drinking criteria before treatment [28].

It was surprising to find patient experience scores were not significantly different in those using and not-using enemas. The authors anticipated that those using enemas would report a worse experience, based on frequent patient disquiet and hesitation witnessed when enemas are proposed. A limitation of the results is that patients only experienced one preparation regime and knew no different option. Implementation of a crossover trial [29], where each patient follows both preparation regimes at different times, would enable individuals to compare regimes. However, this methodology would be impractical, complicated to introduce, and could increase internal anatomy variability during RT.

Study results are also limited by the marked reduction in completed surveys from planning through to treatment. Reasons for the survey not being completed were not formally captured, but treatment radiographers reported incidences where they forgot to give surveys out, or the patient expressed a wish not to complete them. To prompt survey distribution by the treatment teams, all patients completing the baseline scanning survey, had “please give preparation survey” added to their Mosaiq (Elekta, Sweden) treatment calendar at timepoints two and three. Prior to go live, treatment teams were invited to an information session on the project, including survey dissemination timepoints and dissemination method, with a summary email circulated to all. Despite this communication and engagement with the team some surveys were not distributed. Reassigning during treatment survey distribution from the busy treatment unit to a dedicated researcher may have improved completion rates.

Patients’ reluctance to complete the survey during treatment may have been due to questionnaire fatigue, resulting from repeating the activity too frequently. On reflection it is still felt that the survey timepoints implemented were appropriate, however if repeating there is scope for the survey to be shortened, to reduce patient effort and time burden. Patient characteristics such as health status, education and ethnicity may also have impacted individuals’ likelihood of completing the survey, but the limited patient demographic data collected means we cannot assess for this bias.

The proportion of patients having difficulty inserting enemas rose from 18 % at the planning scan to 35 % at the final week of RT. Difficultly inserting enemas can be impacted by many different factors including reduced flexibility and hand dexterity, haemorrhoids, past trauma and anxiety. The rise in difficulty towards the end of treatment suggests that anorectal RT toxicity [18], [19], also evident in our EPIC score results, increased insertion difficulty. Interestingly discomfort inserting enemas did not increase with advancing timepoints, as might have been expected.

This study was not designed to capture why patients found insertion or other aspects of preparation difficult nor the difference in difficulty and discomfort experienced. A further qualitative study, with patient interviews, is planned to gain insight into patient’s experience of preparation and reasons for varied experiences. Results of such a project will be coupled with ongoing work examining the impact of enema/no enema preparation regimes on prostate inter and intrafraction motion to help inform personalisation of preparation regimes.

Anxiety levels associated with preparation (question 4) were highest in the first week of RT, with 26 % of respondents reporting anxiety. Rates were 12 % and 8 % for the planning scan and final week of RT respectively. These results are incongruent to previous literature reporting that the highest levels of anxiety occur pre-treatment [4], [30], cross study comparison is however complicated by varying measurement tools and timepoints.

The authors postulate that the reason preparation anxiety peaked during the first week of RT, is twofold. The first, because of an information gap at this timepoint. As described, current preparation advice is given prior to the planning scan, occurring 2–3 weeks before RT start, during which time individuals may forget or confuse guidance given. An information appointment one day before or on day one of RT is scheduled for all patients with the treating radiographer team, preparation is discussed but the meeting content is not preparation specific, and information is only given verbally. The hope is to reduce week one preparation anxiety by introducing consistent reiteration and reassurance about preparation at this stage. To support this, a guidance document has been produced for radiographers to follow when delivering this information.

The second factor potentially at play is fear associated with radiation treatment itself. Radiotherapy is often unknown to patients before it is recommended to them [31], with some having a negative association with the broader concept of radiation [32], [33]. Fears of RT quality and safety are also reported [34], [35] and may have a greater impact at the beginning of RT, before patients get more used to treatment. The authors recognise that wider public education on radiotherapy is needed to improve public awareness and perception of RT. Future projects will be designed collaboratively with patients and the public to ensure that information given, and questions asked are understood, accessible and relevant to those affected.

An increase in anxiety and depression in the first week of RT was not seen when measured using PHQ4. Mild and worse anxiety and depression affected 13 % of respondents at planning CT and first week of RT, dropping slightly to 12 % in the final week of RT. These rates are higher than the UK male average [36], unsurprising as anxiety and depression are some of the most common symptoms experienced by cancer patients [30]. However, they are slightly lower than published results reporting anxiety prevalence of 27 %, 15 % and 18 %, pre-treatment, on-treatment and post treatment respectively [30] and a depression rate of 17–20 % [37], [38] for prostate cancer patients. This may reflect the quality of preparation advice provided but could also be influenced and limited by the PHQ4, which is an anxiety and depression indicator rather than diagnostic tool. In clinical practice the distress thermometer, validated to detect cancer-specific distress, anxiety and depression is utilised instead [39].

## Conclusion

This work has allowed the authors to better understand patient’s experience preparing for PCa RT. It concludes that the preparation needs of patients are well met, realised by delivery and reiteration of information via a patient seminar, RT consent and a telephone follow-up appointment prior to planning scan. The time delay from planning scan to starting treatment (2–3 weeks), and the lack of consistent preparation specific information given during this wait may have contributed to a peak in preparation anxiety during the first week of RT.

Patient experience of preparation, PHQ4 and question 15 of EPIC scores were not significantly different in those using or not using enema preparation for PCa RT. Despite most patients having a positive preparation experience, a considerable number did find preparation difficult, disruptive and ineffective. With difficulty maintaining a full bladder and using enemas greatest at the end of treatment, likely due to RT toxicity. A qualitative study, utilising patient interviews, is needed to better establish the patient’s experience and understand why variations present. With the aim of using the patient perspective to personalise RT preparation.

## Declaration of competing interest

The authors declare the following financial interests/personal relationships which may be considered as potential competing interests:

*S.E. Alexander:* Cancer Research UK Programme Grant C33589/A28284.

*U. Oelfke*: CRUK Program Grant, Adaptive Data-Driven Radiation Oncology, C33589/A28284.

*H.A. McNair:* Royal Marsden Cancer charity.

*A.C. Tree:* Institution research funding from Elekta, Accuray & Varian.
