# Supplementary figures and images for: Patient experience preparing for prostate cancer radiotherapy
Source: Tech Innov Patient Support Radiat Oncol. 2025 Feb 17;33:100306. doi: 10.1016/j.tipsro.2025.100306 (PMC11905849; doi:10.1016/j.tipsro.2025.100306)

**Supplementary material A**

*Patient preparation survey*


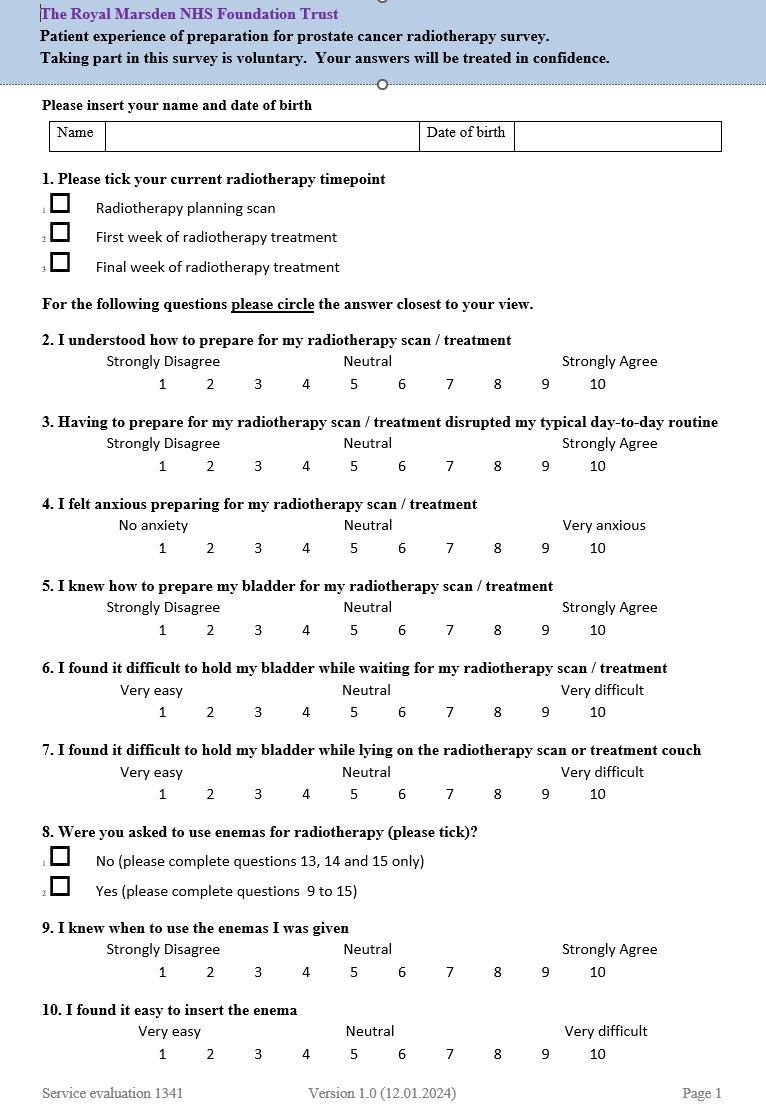


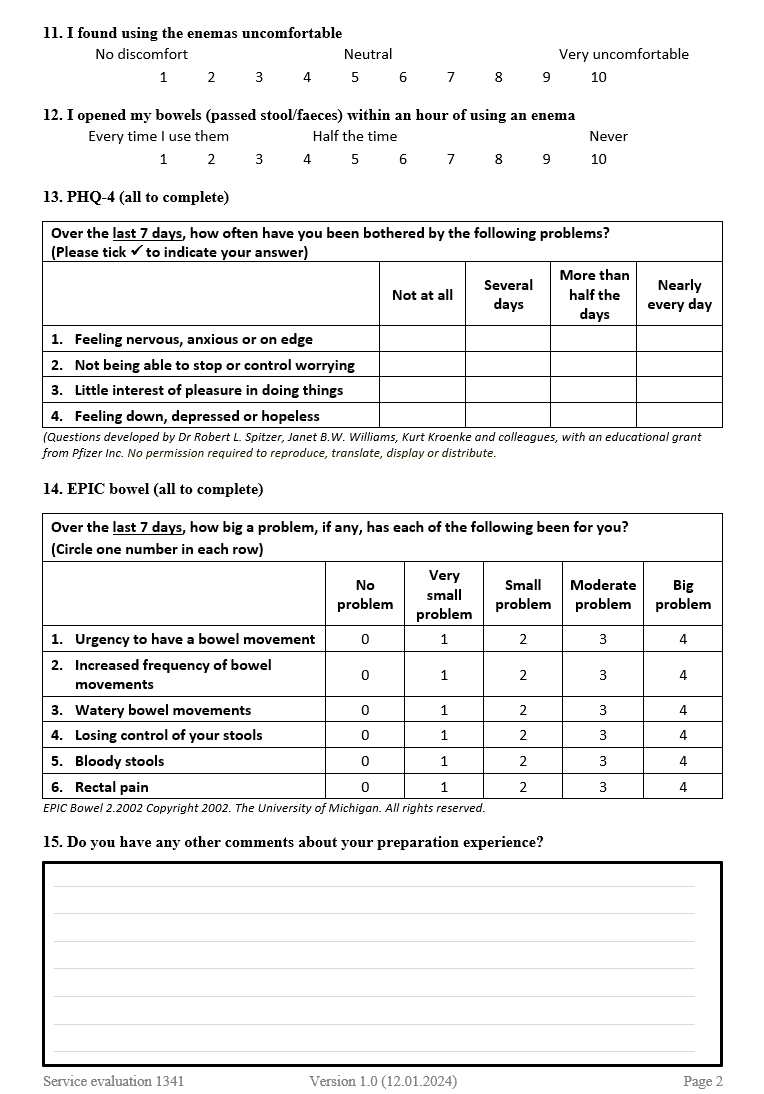

Supplement: Supplementary Data 1 [file mmc1.docx]
